# Robust Smart Superhydrophobic Cilia

**DOI:** 10.1002/advs.202524211

**Published:** 2025-12-16

**Authors:** Chuanqi Wei, Baixue Li, Yimeng Zhang, Oleg V. Gendelman, Youhua Jiang

**Affiliations:** ^1^ Department of Mechanical Engineering (Robotics) Guangdong Technion – Israel Institute of Technology Shantou Guangdong 515063 China; ^2^ Faculty of Mechanical Engineering Technion – Israel Institute of Technology Haifa 3200003 Israel

**Keywords:** magneto‐responsive, robust, smart structures, superhydrophobic, wires

## Abstract

As inspired by natural cilia, smart (stimuli‐responsive) artificial microwires can manipulate objects and tune surface properties, leading to various applications. However, artificial microwires operate merely in gentle working conditions due to their inherent fragility against mechanical damage, indicating that there is a critical gap between the microwires demonstrated in a laboratory and the microwires that operate in practical conditions. This inherent limitation is circumvented by introducing a new surface design, where the microwires are hidden in interconnected frames when facing mechanical damage and can be aroused by external magnetic fields to deliver functionalities when needed. Namely, under a magnetic field parallel to a substrate textured with vertical frames, iron‐laden polydimethylsiloxane (PDMS) aerosols are aligned perpendicularly onto frame sidewalls, forming multilayer microwires parallel to the substrate. The frames prevent mechanical damage from contacting the wires, and the lower layers of wires are functional, although the top layers are worn off, rendering the mechanical robustness. By applying a magnetic field, the wires can re‐align uprightly and hence expose themselves to the working environment, delivering functionalities such as on‐demand control in droplet impact dynamics, adhesion force, and transport. This design strategy paves the way for the utilization of smart surfaces in real‐life conditions.

## Graphical abstract

To date, ciliated surfaces cannot withstand mechanical damage because cilia/wires are inherently fragile. By aligning magneto‐responsive, flexible, superhydrophobic microwires on the sidewall of vertical frames, the frames act as armors to protect microwires from mechanical damage; when needed, the microwires re‐align uprightly under a magnet field to manipulate surface properties, being the first smart ciliated surface that withstands mechanical damage.

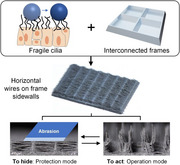

## 1 Introduction

**Figure 1:**
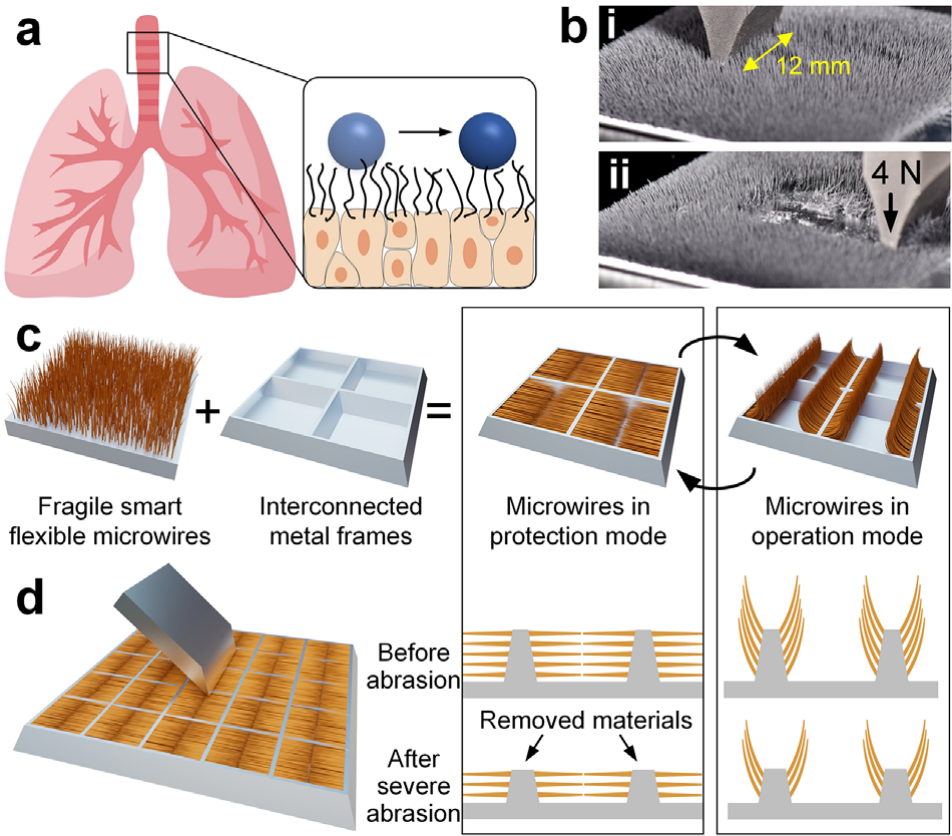
Design of mechanically robust smart cilia. a) Schematic showing object transportation by respiratory cilia. b) Physical damage made to artificial microwires by abrasion at a normal load of 4 N. c) Schematics showing the design strategy of robust microwires by implanting multilayer microwires perpendicularly onto the vertical sidewalls of interconnected frames, where the protection and operation modes of the microwires can be switched by a magnetic field in an on‐demand fashion. d) In the protection model, the interconnected metal frames prevent the grinding head from touching the microwires, and the lower layers of microwires can still operate after severe abrasion. In the operation mode, the horizontal microwires are aroused by a magnetic field to deliver cilia‐related functionalities.

Nowadays, attention in surface/interface science has been switched from how passive surface properties (intrinsic wettability and microstructure morphology) affect droplet‐surface interaction (e.g., contact angles, adhesion, friction, and impact dynamics) to how the controlled motion of active microstructures dictates droplet behaviors (e.g., the manipulation/tunability in droplet adhesion, motion, and impact dynamics). As inspired by respiratory cilia (**Figure** **1**a),^[^ ^1^, ^2^, ^3^ ^]^ various artificial smart (stimuli‐responsive) microwires have been developed to tune surface wettability and manipulate objects, serving various applications such as microfluidics, object manipulation, water‐repelling and anti‐icing surfaces, etc.^[^ ^4^, ^5^, ^6^, ^7^, ^8^, ^9^, ^10^, ^11^, ^12^, ^13^, ^14^, ^15^, ^16^, ^17^, ^18^, ^19^, ^20^, ^21^, ^22^ ^]^ Taking a flat surface decorated with magneto‐responsive flexible microwires (un‐armored ciliated surface) as an example (Figure 1b‐i),^[^ ^8^, ^9^, ^10^ ^]^ the artificial microwires barely survived from one abrasion (a normal load *F*_↓_ of 4 N) because of the inherent fragility of microwires (Figure 1b‐ii). Thus, one encounters a critical and long‐standing gap between the artificial smart microwires demonstrated in the laboratory conditions and those applicable to real‐life operation (where mechanical damage is pervasive).

Although much progress has been made to improve the mechanical robustness of nano‐/microtextured surfaces,^[^ ^23^, ^24^, ^25^, ^26^, ^27^, ^28^, ^29^ ^]^ as far as the authors know, no surfaces with smart and soft microwires have been reported to withstand a substantial abrasion. Our suggestion to cope with the inherent fragility of microwires was to re‐generate the damaged wires, much similarly to the re‐growth of hairs,^[^ ^30^ ^]^ but additional steps for regeneration are required. In this study, we suggest overcoming microwire fragility by hiding magneto‐responsive superhydrophobic microwires in metal frames (serving as armors) when facing mechanical damage and arousing them for operations when needed (Figure 1c). The integration of the mechanical robustness of the frames and the stimuli‐responsiveness of flexible microwires results from a unique microwire‐surface macrostructure configuration. Namely, the frames are perpendicular to the surface substrate, whose sidewalls are normally implanted with multilayer microwires, and hence the microwires are parallel to the surface substrate. The preservation of surface functionalities from mechanical damage follows two mechanisms (Figure 1d): 1) the frames serve as armor preventing the source of abrasion (e.g., a grinding head) from entering the frame pockets that house the microwires; 2) the lower layers of microwires are still functional when those on upper layers are damaged along with the abraded frames. When needed, the horizontal magneto‐responsive wires hidden among frames re‐align uprightly under a magnetic field, exposing themselves to the working environment to deliver functionalities. The hide‐and‐arousal switch of microwires is implemented in an on‐demand fashion.

## 2 Results and Discussion

### 2.1 Demonstration of Surface Mechanical Robustness

**Figure 2:**
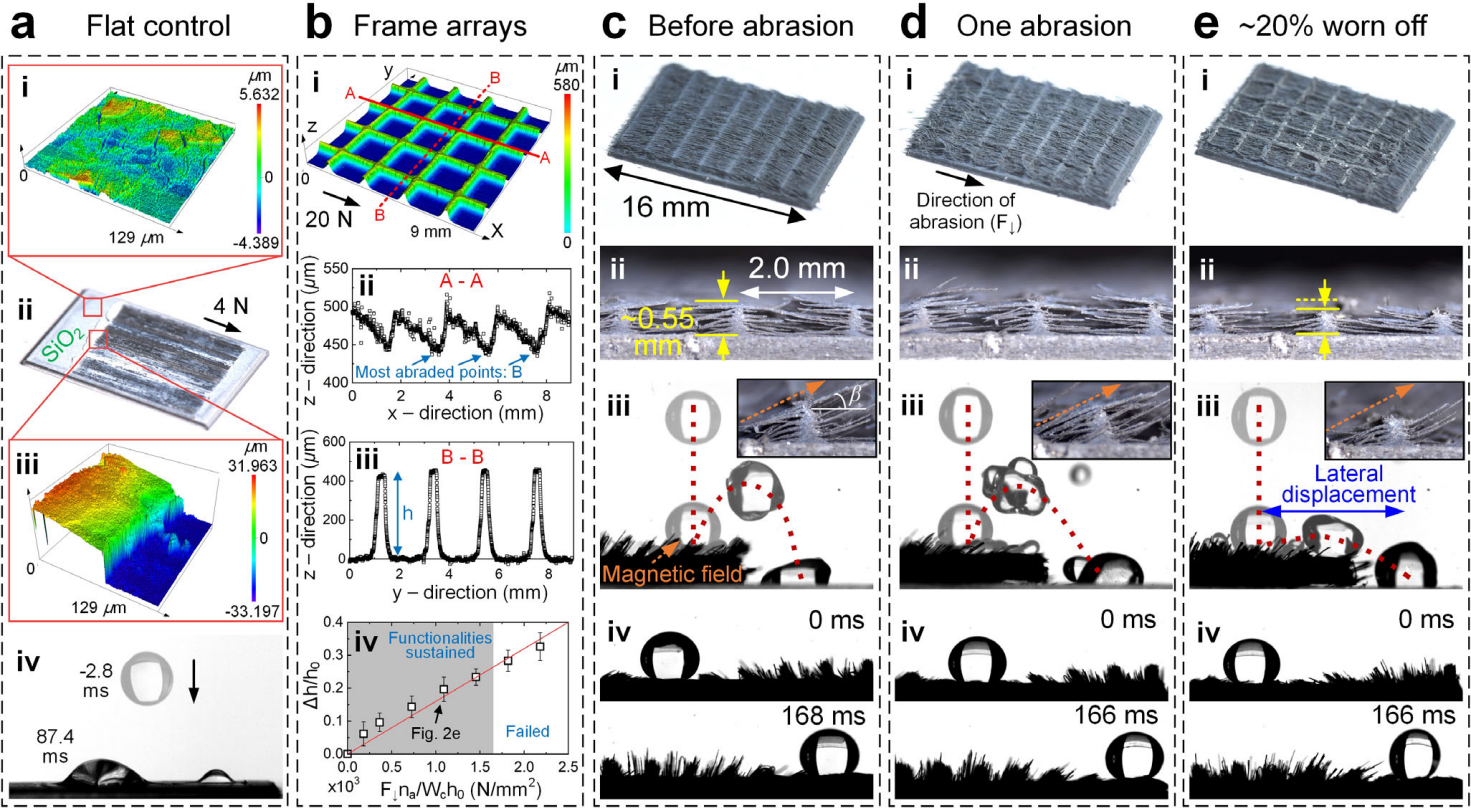
Abrasion test of robust smart cilia and their preserved functionalities. a) Surface morphology of a SiO_2_ nanoparticles‐coated flat surface i) before and ii, iii) after abrasion at a normal load *F*_↓_ of 4 N and iv) a pinned droplet on the damaged area. b) Morphology of frame arrays after being abraded *n_a_* = 12 times at *F*_↓_ of 20 N; iv) the abraded percentage of frame sidewall heights Δ*h*/*h*_0_ is plotted with *F*_↓_ *n_a_*/*W_c_h*_0_, where the fitted slope is wear rate *K*/*H* = 0.16 $mm^{3}N^{-1}m^{-1}$. Robust smart cilia c) before abrasion, d) after one abrasion at *F*_↓_ of 4 N, and e) with ≈20% of frame heights being worn off. Different rows in c–e): surface i) top and ii) cross‐section views, iii) directional bouncing of a 20 *µL*‐water droplet released at 6 cm onto inclined microwires, and iv) transport of a 10 µL‐water droplet by collective motion of microwires.

The physical damage to the surface is twofold: the loss of superhydrophobic coatings and the surface macro‐structures. The surface superhydrophobicity is rendered by hydrophobic SiO_2_ nanoparticles, which can be easily destroyed by one abrasion with *F*_↓_ of 4 N. Specifically, surface morphologies of a flat aluminum substrate coated with SiO_2_ nanoparticles before (**Figure** **2**a‐i) and after (Figure 2a‐iii) abrasion are characterized, where the removal of SiO_2_ nanoparticles is evident. As a result, a droplet remains pinned on the damaged surface (Figure 2a‐iv).

In addition to the physical damage to the superhydrophobic coatings, the frame sidewalls and the attached microwires can be worn off. Since the presence of microwires hinders the accurate measurement of the abraded surface, the abrasion test was conducted using frame arrays without microwires (Figure 2b), where the initial frame sidewall length *w* (*h* = *h*_0_) is 2.0 mm and top sidewall thickness *b* (*h* = *h*_0_) is 0.1 mm. The most abraded points (the points with the lowest height in Figure 2b‐ii) were identified and their decreases from the initial height Δ*h* = *h*_0_  −  *h* were measured for different numbers of abrasions *n_a_* (Figure 2b‐iii). The maximal decrease in frame height Δ*h* follows the Archard equation,^[^ ^30^, ^31^, ^32^, ^33^ ^]^ Δ*hL* *W_c_* =  (*K*/*H*) · *F*_↓_ *n_a_L*, where Δ*hLW_c_* represents the total volume of the abraded materials, *L* = 10 mm is the travel distance of the grinding head per abrasion, *W_c_* approximates the grinding head‐substrate contact area per unit traveling length (*W_c_* = 4*b* in this study as the grinding head contacts four frame sidewalls), *F*_↓_ is 20 N, and *K*/*H* (in a unit of mm^3^ N^−1^m^−1^) is wear rate with *K* being a dimensionless wear coefficient and *H* being the Brinell indentation hardness of the material. All data points collapse onto a straight line when the measured decrease in sidewall height normalized by its initial height Δ*h*/*h*_0_ is plotted with *F*_↓_ *n_a_*/*W_c_h*_0_ (in a unit of N mm^−^^2^), where the slope is wear rate *K*/*H* (Figure 2b‐iv).

The i) top and ii) cross‐section views of robust smart cilia before and after abrasion are shown in Figure 2c–e along with their superhydrophobicity and stimuli‐responsiveness‐related functionalities, such as iii) the droplet self‐removal by directional bouncing and iv) active droplet transport by wire beating (see also Video S1 in Supporting Information). Taking the surface with frame sidewall length *w* of 2.0 mm (wire length *w*/2 of 1.0 mm) as an example, a 20 µL‐droplet (radius *R* of ≈1.68 mm) released at 6 cm (Weber number *We*, $\rho v_{0}^{2}R/\gamma_{LV}$ = 27) demonstrated directional bouncing when the microwires were titled (*β* ≈ 30°) under an inclined magnetic field (Figure 2c‐iii), indicating the surface capability for droplet self‐removal. This is attributed to the imbalanced speed/momentum of the droplet retracting boundary,^[^ ^8^, ^30^, ^34^, ^35^ ^]^ where the contact line recedes at a larger speed in the direction along the wire axial direction than its counterpart. Moreover, a water droplet can be precisely transported following a moving magnet by collective motion of microwires (Figure 2c‐iv),^[^ ^6^, ^10^, ^30^ ^]^ demonstrating the active control of objects for applications such as microfluidics.

After one abrasion at *F*_↓_ of 4 N, in contrast to the loss of microwires on an unarmored substrate (Figure 1b) and the loss of superhydrophobicity (SiO_2_ nanoparticle coatings in Figure 2a), functionalities such as directional bouncing and transport of droplets remain unaffected (Figure 2d), which evidences the survival of microwires and the preservation of surface superhydrophobicity. This is because although the SiO_2_ nanoparticles on frame tops were removed, the preserved superhydrophobic microwires are still sufficient to deliver functionalities. Following the data in Figure 2b‐iv, ≈20% of the frame sidewalls have been worn off after *n_a_* = 12 abrasions at *F*_↓_ of 20 N, while the surface functionalities sustain (Figure 2e‐iii, iv), suggesting an excellent resistance to performance degradation by abrasion.

### 2.2 Characterization of Surface Functionality Degradation

**Figure 3:**
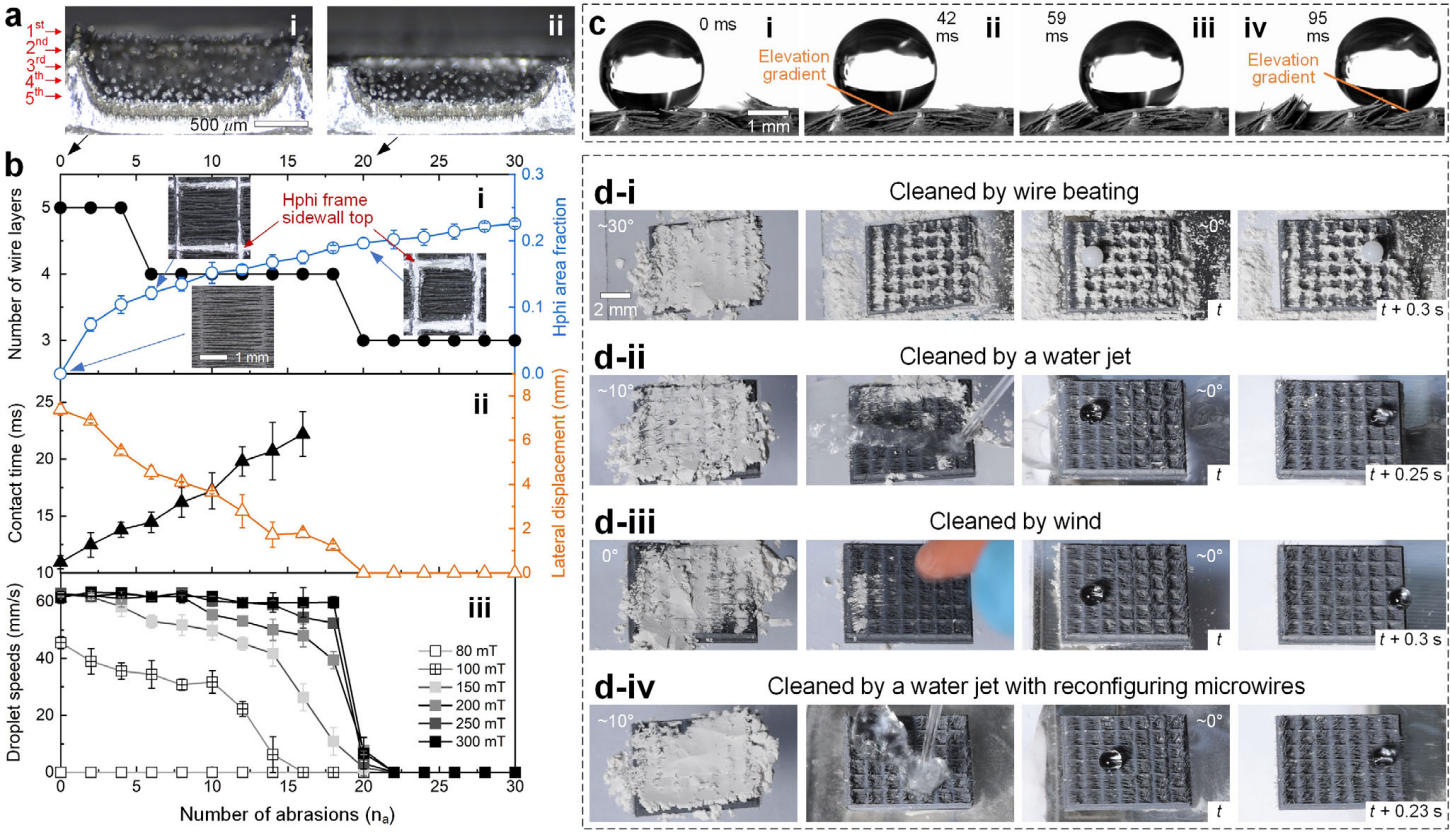
Surface functionality degradation by abrasion and dust‐related pollution. a) cross‐section views of the robust smart cilia i) before abrasion and ii) after *n_a_* = 20 abrasions. b) the variations of surface morphology and functionalities with respect to the number of abrasions: i) the layers of remaining microwires and the measured hydrophilic area fraction, ii) the contact time on surfaces with upright wires and the lateral displacement on surfaces with inclined wires of 20 µL‐droplets released at 6 cm, and iii) droplet speeds when manipulated by magnets with different magnetic field strengths that moved at 60 mm/s. c) Dynamics of microwires for the manipulation of a 10 µL‐water droplet. d) The removal of dust (*µm*‐sized coal ashes) deposited on the robust smart cilia for sphere/droplet manipulation: i) by wire beating, ii) by a water jet, iii) by a gentle wind, and iv) by a water jet combined with varying microwire configurations. The inclination angle of a substrate is denoted. All surfaces have *w* = 2.0 mm.

To systematically study the extent to which the robust smart cilia withstand abrasion and the mechanism of how surface functionalities are preserved, we examine the microscopic morphology of the sample after every two abrasions from the top and cross‐section views (**Figure** **3**a,b). Results show that although the number of remaining layers of microwires decreases with an increase in the number of abrasions *n_a_* at F↓ of 20 N (Figure 3b‐i), the frame pockets (pores) remain covered by microwires while the aluminum frame sidewalls, which are intrinsically hydrophilic (Hphi), are exposed. Because of the pyramidal shape of a pore, the frame pocket length *w* and top wall thickness *b* decrease and increase slightly with a decrease in frame sidewall height *h*, respectively. This leads to an increase in the hydrophilic area fraction of a surface, i.e., the area of frame sidewall top normalized by the projected area of frame sidewall top and pore, 1 − *w* ^2^/(*w* + *b*)^2^, with an increase in *n_a_* (Figure 3b‐i). As a result, the pinning effects of droplets caused by the hydrophilic areas impede the surface functionalities, and to a certain extent, the surface functionalities vanish.

The measured droplet‐substrate contact time *t_ct_* of 20 µL‐droplets released at 6 cm on the abraded surfaces with upright microwires, which were reconfigured by a magnetic field perpendicular to the substrate, increases with an increase in *n_a_* (Figure 3b‐ii). When *n_a_* < 12 (Δ*h*/*h*_0_ < 20%), *t_ct_* are less than that on a flat superhydrophobic surface (≈19 ms, see also Figure 7h‐i).^[^ ^36^ ^]^ This is because the upright microwires allow the penetration of liquids into frame pockets, whose upward retraction with sufficient momentum (proportional to the penetrating liquid mass per pocket) lifts the droplet before its full retraction.^[^ ^30^, ^37^ ^]^ Moreover, the lateral displacement (the distance between the impacting site and the landing site of the first bounce, Figure 2e‐iii) of the same droplets on the surfaces with inclined microwires, which quantifies the surface capability for droplet self‐removal, decreases with an increase in *n_a_* (Figure 3b‐ii). The above results suggest that the remaining microwires can be reconfigured by a magnetic field after abrasion, and the superhydrophobicity provided by the microwires can overcome the pinning effects. However, when *n_a_* exceeds 12 and 20 (corresponding to hydrophilic area fractions of 0.15 and 0.2, respectively), the reduction in contact time (a demonstration of excellent water repellency) and the lateral displacement (an indication of overcoming pinning effects) vanish, respectively (Figure 3b‐ii).

The surface functionality is also represented by the directional manipulation of objects. The detailed dynamics of microwires show that a droplet initially rests on a frame pocket supported by two columns of microwires implanted on opposite sidewalls (Figure 3c; Video S2, Supporting Information). The moving magnet coming from the left makes the left column of microwires move upward (rotate counterclockwise in Figure 3c‐ii), creating an elevation gradient that drives the droplet to move rightward under the effects of gravity. This process repeats for the next frame pocket and hence leads to a directional movement. Since the droplet motion is caused by the surface elevation gradient, which follows the motion of a magnet, the droplet has an identical speed with the magnet up to 100 mm s^−1^ (Figure 3b‐iii).^[^ ^10^ ^]^ When *n_a_* is below a critical value, and the magnetic field strength is larger than 80 mT, the gravitational effects coupled with the elevation gradient overcome the pinning effects at hydrophilic frames, and hence, the droplet can be manipulated. The critical number of abrasions depends on the magnetic field strength in a way that a larger magnetic field strength allows droplet manipulation on a more severely abraded surface (Figure 3b‐iii). This is because the lifting microwires empowered by a larger magnetic field strength have larger momentum to motivate the droplet.

In addition to abrasion, dust‐related pollution and intensive usage of microwires are other main factors leading to functionality degradation. The robust smart cilia are fully covered by a substantial amount of micro‐coal ashes (S95 grade mineral powder), a typical representative of dust (Figure 3d).^[^ ^38^ ^]^ When the surface is tilted at ≈30°, particles can be removed from the top of microwires and frame pockets by wire beating controlled by a magnet. Then, this surface is leveled followed by depositing a 3 mm‐diameter POM (polyoxymethylene) sphere. The sphere can be directionally manipulated despite substantial particles remaining on frame sidewall tops (Figure 3d‐i; Video S3, Supporting Information). This suggests that microwires function well under the effects of dust‐related pollution. One should note that a water droplet cannot be manipulated on such a heavily polluted surface because the droplet remains pinned by wetting the mineral powder. The coated dust can be efficiently removed by a water jet (Figure 3d‐ii) and a gentle wind produced by an ear syringe (Figure 3d‐iii), and can be thoroughly removed by a water jet while varying the wire configurations by a magnet (Figure 3d‐iv). Droplet manipulation can be achieved on the cleaned surfaces, suggesting the surface robustness under dust‐related pollutions. We also confirm that the microwires function well by transporting droplets after 1200 cycles of back‐and‐forth actuation by a magnet without any degradation (Video S4, Supporting Information).

**Figure 4:**
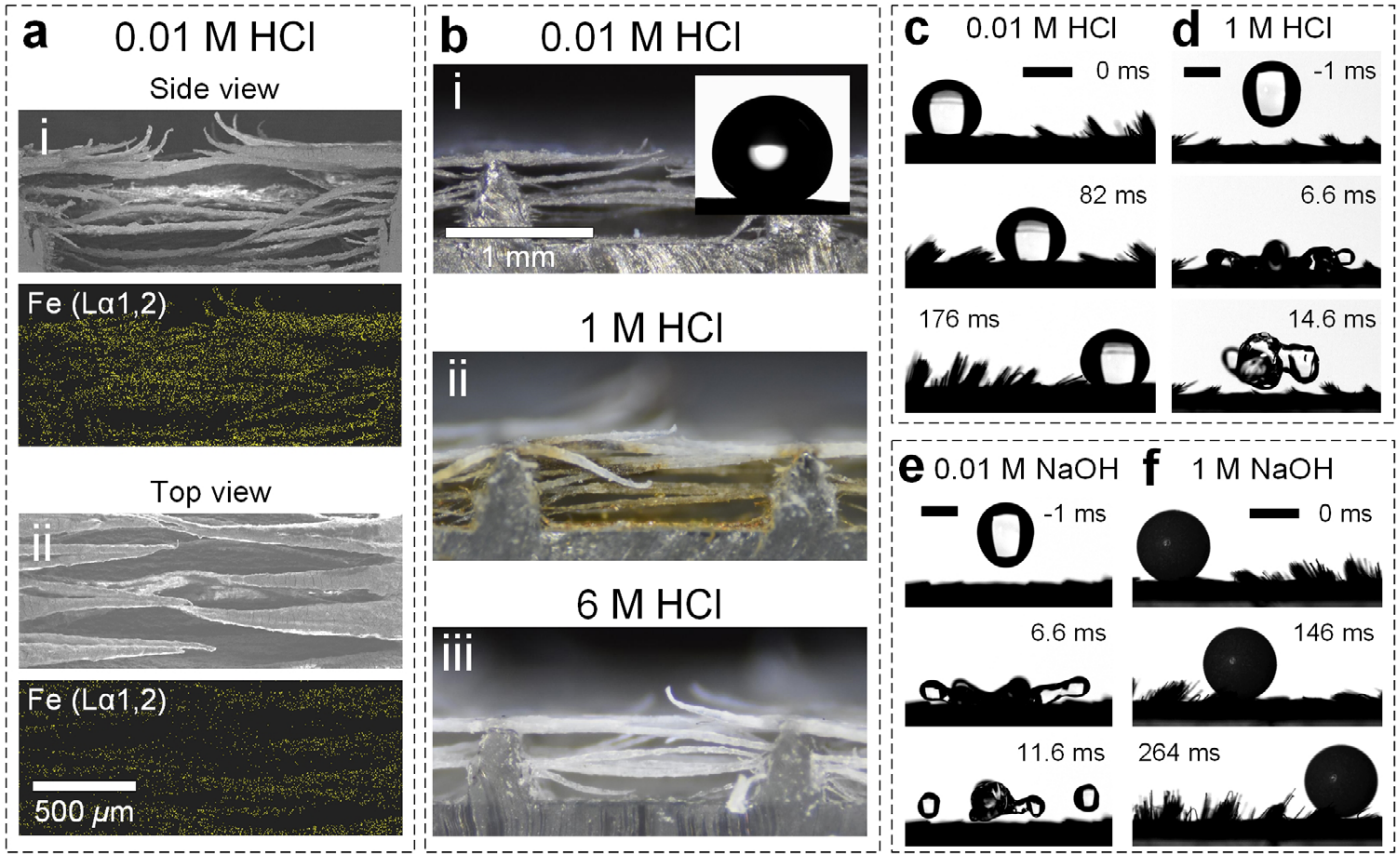
Surface functionality degradation by acid and alkali solutions. a) EDS test of the robust smart cilia from the side and top views with the exhibition of iron (Fe) distribution. b) Optical images of the robust smart cilia after being immersed in i) 0.01 M, ii) 1 M, and iii) 6 M HCl solutions for 24 h. Droplet/sphere manipulation on surfaces that have been immersed in c) 0.01 M HCl and f) 1 M NaOH solutions and droplet impact dynamics on surfaces that have been immersed in d) 1 M HCl and e) 0.01 M NaOH solutions. The scale bars in (c–f) are 2 mm.

Surface degradation can be also caused by acid and alkali solutions. Since iron (Fe) reacts with HCl,^[^ ^10^, ^39^ ^]^ we expect that robust smart cilia will lose magneto‐responsiveness due to the loss of iron particles. However, uniform distribution of iron (Fe) on microwires (**Figure** **4**a) can be observed on the sample immersed in 0.01 m (pH = 2) HCl for 24 h (Figure 4b‐i), and this sample can manipulate a water droplet by a magnet (Figure 4c), suggesting the surface durability against mild acid solutions. In contrast, yellow‐to‐brown color (ferric chloride) can be observed on microwires immersed in 1 m (pH = 0, Figure 4b‐ii) HCl, and transparent microwires can be observed on samples immersed in 6 m HCl (Figure 4b‐iii), suggesting the loss of iron particles. Nevertheless, the water repellency remains intact because HCl does not damage fluorinated coatings,^[^ ^39^ ^]^ supported by a water droplet released at 6 cm jumping from the 1 m HCl immersed sample with a contact time of 14.6 ms (Figure 4d). Alkali solutions are known to damage fluorinated coatings.^[^ ^39^ ^]^ Nevertheless, the same rebouncing droplet can be observed from the robust smart cilia immersed in 0.01 m (pH = 12) NaOH solutions for 24 h (Figure 4e), suggesting the surface resistance to mild alkali solutions. The sample immersed in 1 m (pH = 14) NaOH solutions loss water repellency, whereas the magneto‐responsiveness remains, indicated by the manipulation of a 3 mm‐POM sphere (Figure 4f).

### 2.3 Fabrication Processes and Surface Morphology Control

**Figure 5:**
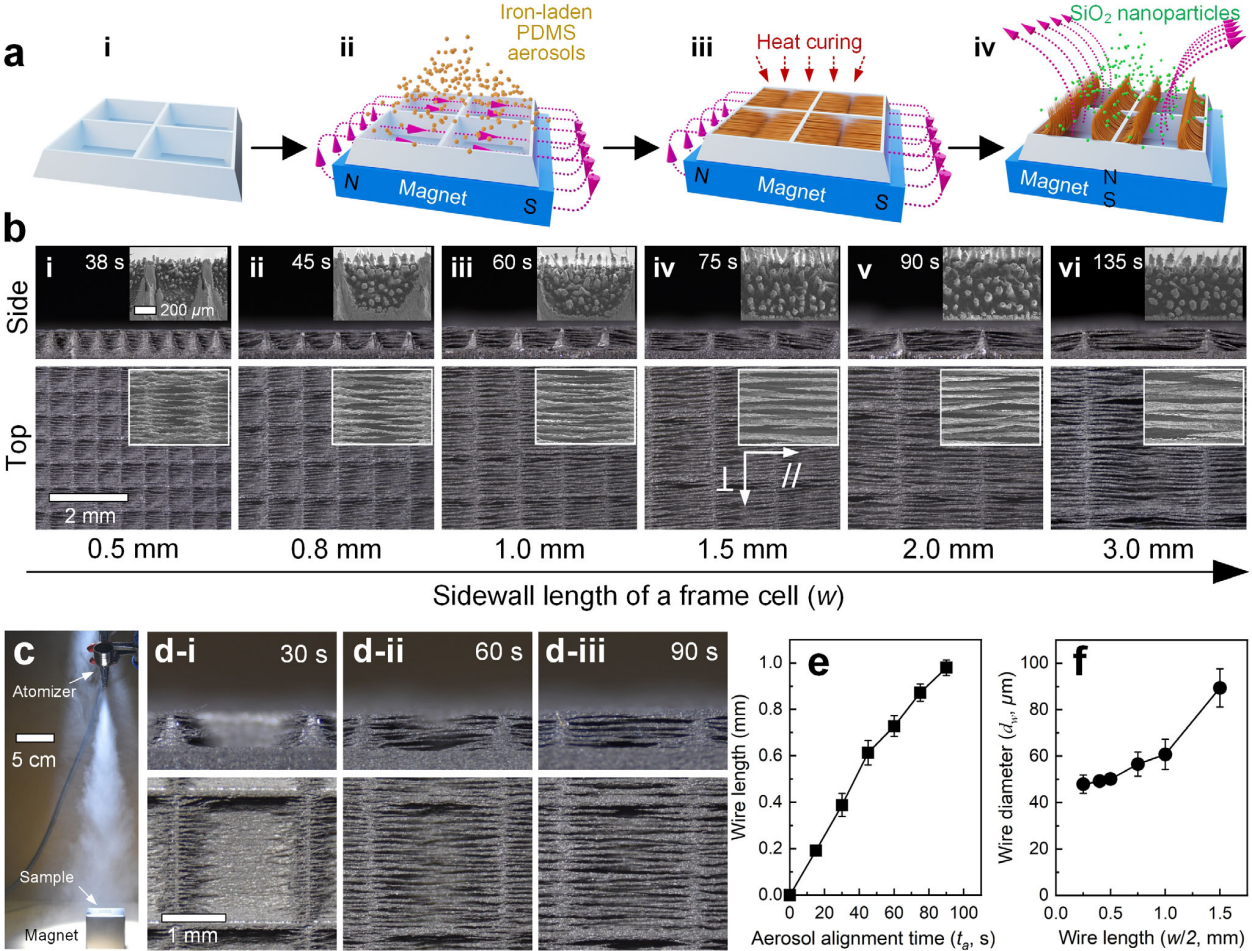
The fabrication of robust smart cilia. a) Fabrication process: i) laser‐texturing of an aluminum plate for (sub‐)millimetric interconnected frames; ii) alignment of iron‐laden aerosols perpendicularly onto frame sidewalls under a magnetic field in parallel to the surface substrate followed by iii) heat curing; iv) the coating of hydrophobic SiO_2_ nanoparticles onto upright wires under a magnetic field for surface super‐hydrophobization. b) Morphology of the fabricated robust smart cilia with different wire and frame sidewall lengths, where the corresponding aerosol alignment times *t_a_* are denoted. Since microwires are aligned in one direction, the symbols *∥* and ⊥ represent the droplet motion direction in parallel and perpendicular to the wire axial direction, respectively. The inset shows the SEM images of the microwires. c) The real‐time experimental process for the growth of iron‐laden wires. For *w* = 2.0 mm, d) surface morphology and e) microwire length at different *t_a_*. f) Measured microwire diameter *d_w_* at different wire lengths.

An aluminum substrate textured with interconnected frames (pyramidal pores) is placed above a magnet with magnetic field lines parallel to the substrate (**Figure** **5**a‐i). Iron‐laden polydimethylsiloxane (PDMS) solutions are atomized at a rate of 0.03 mL s^−1^ toward the substrate, and the iron‐laden aerosols align following the magnetic field lines (Figure 5a‐ii,c), forming iron‐laden microwires implanted perpendicularly onto frame sidewalls (Figure 5a‐iii). After heat‐curing, the surface with upright microwires under a magnetic field was super‐hydrophobized by coating hydrophobic SiO_2_ nanoparticles (Figure 5a‐iv).

The top and cross‐section views of the robust smart cilia with *w* of i) 0.5 mm, ii) 0.8 mm, iii) 1.0 mm, iv) 1.5 mm, v) 2.0 mm, and vi) 3.0 mm are shown in Figure 5b along with the aerosol alignment times *t_a_* (38 to 135 s). The alignment times have been tuned to ensure the microwires fully cover the frame pockets while the microwires have free ends, i.e., wire length is *w*/2. Specifically, Figure 5d exhibits the surface morphology of *w* = 2.0 mm at *t_a_* of 30, 60 and 90 s, corresponding to a growth rate of microwires at ≈10 µm s^−1^ (Figure 5e). Frame pockets with *w* < 0.5 mm were not adopted in this study because microwires are too short to be substantially manipulated. The SEM images suggest that the center‐to‐center spacing λ_*w*_ between adjacent microwires is ≈0.1 mm and the microwire diameter *d_w_* increases slightly with *t_a_*. For *w*/*2* ≤ 0.75 mm, *d_w_* are ≈50 µm. And *d_w_* are ≈60 and ≈90 µm for *w*/*2* = 1.0 and 1.5 mm, respectively (Figure 5f). Hence, the length‐to‐diameter aspect ratio *w*/2*d_w_* ranges from 5 to 17.

### 2.4 Surface Characterization Using Droplet Adhesion

The extent to which a surface repels water, which is essential to various surface functionalities, can be characterized by the force that laterally de‐pins the droplet, that is, the droplet lateral adhesion force, as

(1) $$F=k\gamma_{LV}D_{b}\left(\cos\theta_{Rear}-\cos\theta_{Front}\right)$$

where *D_b_* is the characteristic droplet base width/diameter, θ_*Rear*_ and θ_*Front*_ are apparent contact angles at the droplet rear and front edges, respectively. The values of θ_*Rear*_ and θ_*Front*_ are close to the droplet apparent receding θ_*R*_ and advancing θ_*A*_ contact angles, respectively, because the rear edge recedes and the front edge advances. The value of *k*, which has been reported to vary from 0.5 to *π*/2,^[^ ^40^, ^41^, ^42^, ^43^, ^44^ ^]^ accounts for the shape of the droplet base, the anisotropy and synchronicity of the moving contact line, and how the contact angle distributes along the droplet base perimeter.^[^ ^44^, ^45^, ^46^, ^47^ ^]^ Since the specific value of *k* remains an open question and deserves independent studies, following recent studies,^[^ ^44^, ^48^ ^]^ *k* is taken as 1 for the sake of simplicity because *k* was measured to be 0.88 ± 0.2 using various liquid/surface combinations.^[^ ^48^ ^]^

**Figure 6:**
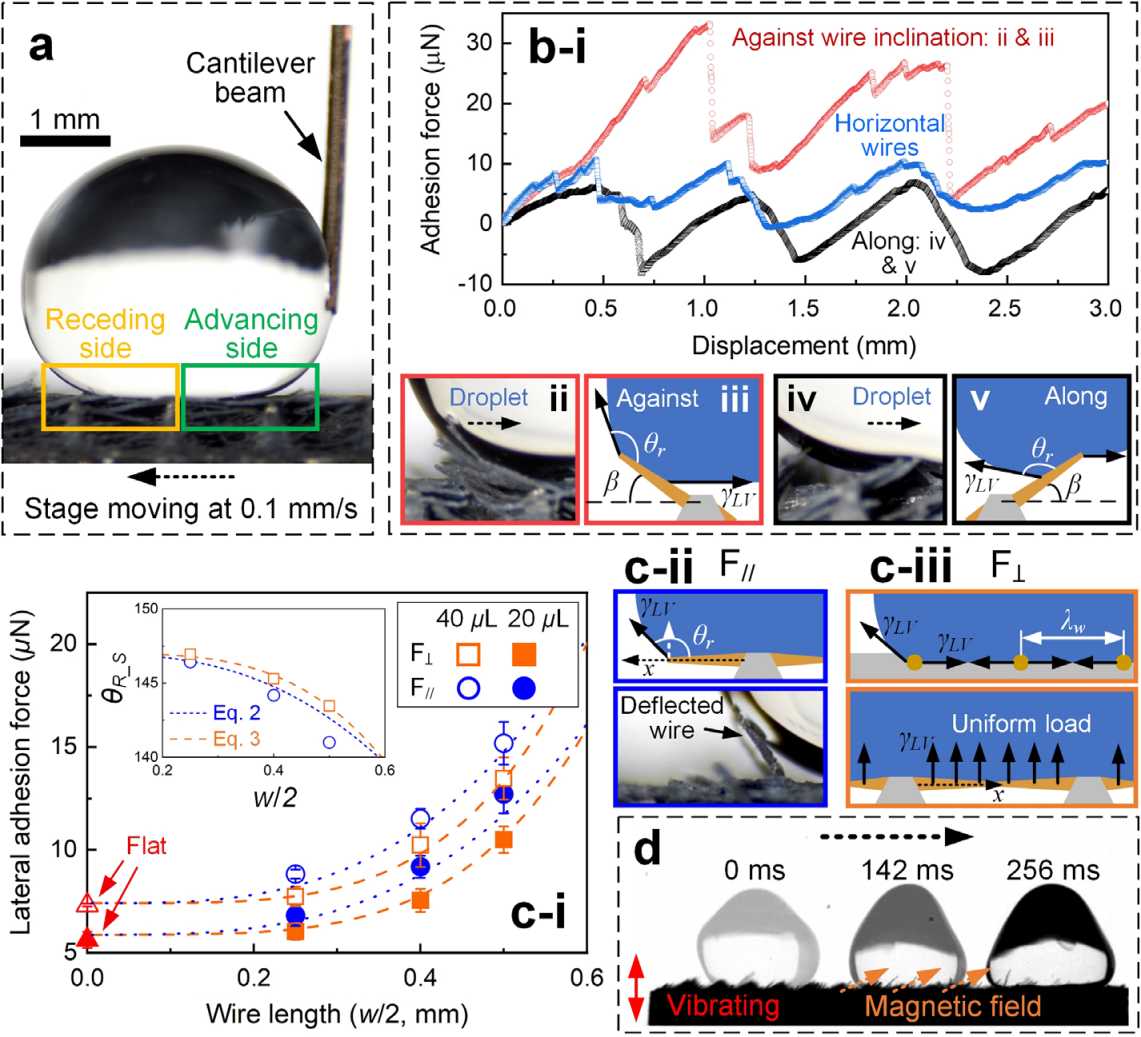
Surface characterization using droplet adhesion. a) Schematics showing the measurement of the droplet lateral adhesion force. b) Measured droplet adhesion force parallel to the wire axial direction *F*_∥_ with respect to surface displacement on substrates with horizontal wires (without a magnetic field) and inclined wires (deflection angle *β*) under a magnetic field. c) The measured (dots) and predicted (lines, Equation 1) maximal adhesion force in parallel *F*_∥_ and perpendicular *F*_⊥_ to wire axial direction on substrates with varying wire lengths *w*/2 for droplets with 20 and 40 µL. Inset: the measured (dots) and predicted (lines, Equations 2 and 3) droplet receding contact angles observed in parallel and perpendicular to the wire axial direction with respect to *w*/2. Schematics showing the capillary forces acting on microwires when the droplet moves ii) in parallel and iii) perpendicular to the wire axial direction. d) Droplet directional motion on a vertically vibrating substrate with inclined wires under a magnetic field.

Since microwires are aligned in parallel to the substrate with only one axial direction, the droplet may experience different adhesion forces when moving in parallel (*F*_∥_) or perpendicular (*F*_⊥_) to the wire axial directions (Figure 5b‐iv). The lateral adhesion force of a 20 µL‐droplet is measured using the tip deflection of a cantilever beam as the droplet slides along the moving sample (**Figure** **6**a). To minimize the geometrical effects of millimetric frame pockets (*D_b_* > *w*) and adopt an assumption of small beam deflection of microwires for analysis, surfaces with *w* = 0.5, 0.8, and 1.0 mm were tested (the measured *d_w_* is 0.05 mm, Figure 5f). Taking the droplets sliding along the wire axial direction as an example, the measured forces *F*_∥_ on the surface with *w* = 0.8 mm increase until a maximal value is reached and then decreases to a minimal value (blue dots in Figure 6b‐i); this increase‐and‐decrease cycle (stick‐slip behavior) repeats, and each cycle corresponds to the movement across a frame pocket. The sudden decrease in force is attributed to the de‐pinning of the pinned contact line and the gravity‐driven movement of the droplet toward the frame pocket center. The maximal values are taken as the measured lateral adhesion forces, which are all larger than that on a superhydrophobic flat control and increase with an increase in wire length *w*/2. Moreover, *F*_∥_ are systematically larger than *F*_⊥_ (dots in Figure 6c‐i).

It is suggested that the microscopic advancing contact angle approaches 180° on superhydrophobic surfaces because the liquid‐vapor interface descends onto the next arrays of structures while the contact line remains pinned.^[^ ^49^, ^50^, ^51^ ^]^ Hence, θ_*Front*_ and θ_*A*_ are indifferent to surface geometrical dimensions, and their apparent values were measured to be 152° ± 2°. The receding (de‐wetting) contact line was regarded as the main reason determining the droplet adhesion force.^[^ ^49^, ^50^, ^51^, ^52^, ^53^, ^54^ ^]^ Since the geometrical effects of millimetric frame pockets are minimized and all surfaces were super‐hydrophobized by the same procedure, the variations of lateral adhesion force with wire lengths and droplet motion directions (*F*_∥_ vs *F*_⊥_) are attributed to the elastic deformation of microwires,^[^ ^55^, ^56^, ^57^ ^]^ which are reflected by the variations in θ_*R*_ (= $\theta_{R\_S}$, where the subscript *S* denotes soft).

Assuming the droplet receding regime adopts a spherical‐cap shape with a receding contact angle θ_*r*_ (147° ± 2°) equal to that measured on a super‐hydrophobized flat control, *D_b_* of the receding regime is 2[3*Vsin*θ_*r*_(1 + *cos*θ_*r*_)/(π(1 − *cos*θ_*r*_)(*cos*θ_*r*_ + 2))]^1/3^. The de‐wetting energy of an apparent droplet contact line on a soft superhydrophobic substrate is the de‐wetting energy per unit area $\gamma_{LV}\cos\theta_{R\_S}$ multiplying the apparent area by which the apparent contact line slides λ*N*Δ*x*,^[^ ^50^, ^55^, ^58^ ^]^ where λ*N* is the apparent receding contact line length, λ (= *w* + *b*) is the center‐to‐center spacing of frame pockets, *N* is the number of frame pockets covered by the receding contact line, and Δ*x* is an imaginary contact line displacement. By assuming the surface covered by dense horizontal microwires is macroscopically flat, the apparent de‐wetting energy $\gamma_{\mathrm{LV}}\cos\theta_{R\_S}\cdot \lambda N\Delta x$ originates from the de‐wetting energy on a flat surface γ_*LV*_cosθ_*r*_ · λ*N*Δ*x* and the elastic energy *NE_E_* (the subscript *E* denotes elastic) of deformed microwires. For contact lines moving in parallel (∥) and perpendicular (⊥) to the wire axial directions, *E_E_* is expressed as $ne_{e}^{∥}$ and $2e_{e}^{⊥}$, respectively, where *e_e_* is the elastic energy per microwire. This is because *n* ≈ *w*/λ_*w*_ wires per frame pocket are deflected in the parallel case by a point force acting on the wire tip *f*  = γ_*LV*_ *wsin*θ_*r*_/*n* (Figure 6c‐ii) with the bending moment *M*(*x*) of − *f*(*w*/2 − *x*). As for the perpendicular case, two wires per frame pocket are assumed to be deflected by a uniformly distributed load $q=\gamma_{LV}\;\sqrt{2+2cos\theta_{r}}$ sourcing from the liquid‐vapor interfaces above and in between wires (Figure 6c‐iii), and the bending moment *M*(*x*) is *q*(*w*/2 − *x*)^2^/2. By solving $e_{e}=\int_{0}^{w/2}\left[M{\left(x\right)}^{2}/2EI\right]dx$ and dividing γ_*LV*_λ*N*Δ*x* at both sides,^[^ ^55^ ^]^ $\theta_{R\_S}$ when moving in parallel and perpendicular to the wire axial directions can be estimated using frame size *w*, frame spacing *λ*, Young's modulus *E* (6 MPa for PDMS) of microwires, and wire area moment of inertia *I* = $\pi d_{w}^{4}/64$, respectively, as

(2) $$cos\;\theta_{R\_S}^{∥}=\;\cos\theta_{r}+\frac{\gamma_{LV}w^{5}\sin^{2}\theta_{r}}{48nEI\lambda \Delta x}$$

and

(3) $$cos\;\theta_{R\_S}^{⊥}=\;\cos\theta_{r}+\frac{\gamma_{LV}w^{5}\left(1+\cos\theta_{r}\right)}{320EI\lambda \Delta x}\;$$

Here, the imaginary contact line displacement, which can also be treated as a fitting coefficient, is taken as the minimal size of the frame (Δ*x* = 0.5 mm). To corroborate Equations 2 and 3, the measured (dots) and predicted (lines) $\theta_{R\_S}^{∥}$ and $\theta_{R\_S}^{⊥}$ are compared in the inserted image of Figure 6c‐i, showing good agreement. Since *D_b_* can be approximated using droplet volume *V* (20 µL and 40 µL) and θ_*r*_ = 147° and θ_*Front*_ is taken as 152°, inserting Equations 2 and 3 into Equation 1, droplet adhesion forces when moving in parallel (*F*_∥_) and perpendicular (*F*_⊥_) to the wire axial directions can be also estimated (lines in Figure 6c‐i). The agreement between the measured (dots) and estimated (lines) values confirms that the increase of adhesion force with an increase in *w/2* is attributed to microwire deformation.

When a magnetic field is inclinedly applied to the surface, microwires are inclined at an angle *β* ≈ 30° (Figure 6b‐ii to 6b‐v). This asymmetric surface configuration leads to an observation that $F_{∥}^{along}$ (when moving along the wire inclination, black dots) is smaller and $F_{∥}^{against}$ (when moving against the wire inclination, red dots) is larger than *F*_∥_ (Figure 6b‐i), which can also be explained by wire deflection. The force component that deflects a wire in the case of $F_{∥}^{against}$ is γ_*LV*_ *w*(*sin*β + *sin*θ_*r*_)/*n* (Figure 6b‐iii) and that in the case of $F_{∥}^{along}$ is γ_*LV*_ *w*|*sin*β − *sin*θ_*r*_|/*n* (Figure 6b‐v), being larger and smaller than that (γ_*LV*_ *wsin*θ_*r*_/*n*) for horizontal wires, respectively. As a result, a droplet moving along the wire inclination causes less wire deflection than its counterpart, and hence $F_{∥}^{along}$ < $F_{∥}^{against}$ (Figure 6b‐i), suggesting a tunability in droplet adhesion force.

As a result, when a vertical vibration (frequency of 100 Hz and amplitude of 0.1 mm) is applied to the surface with inclined wires, transport of a 20 µL‐droplet toward the direction of wire inclination is observed (Figure 6d; Video S5, Supporting Information). The vibrating droplet undergoes a periodical advancing and receding motion. Due to the anisotropy in adhesion force (Figure 6b), the droplet boundary recedes more in the direction along the wire inclination than its counterpart; the accumulation of this disparity in periodical motions leads to droplet directional transport.^[^ ^59^, ^60^, ^61^ ^]^

### 2.5 Surface Characterization Using Droplet Impact Dynamics

**Figure 7:**
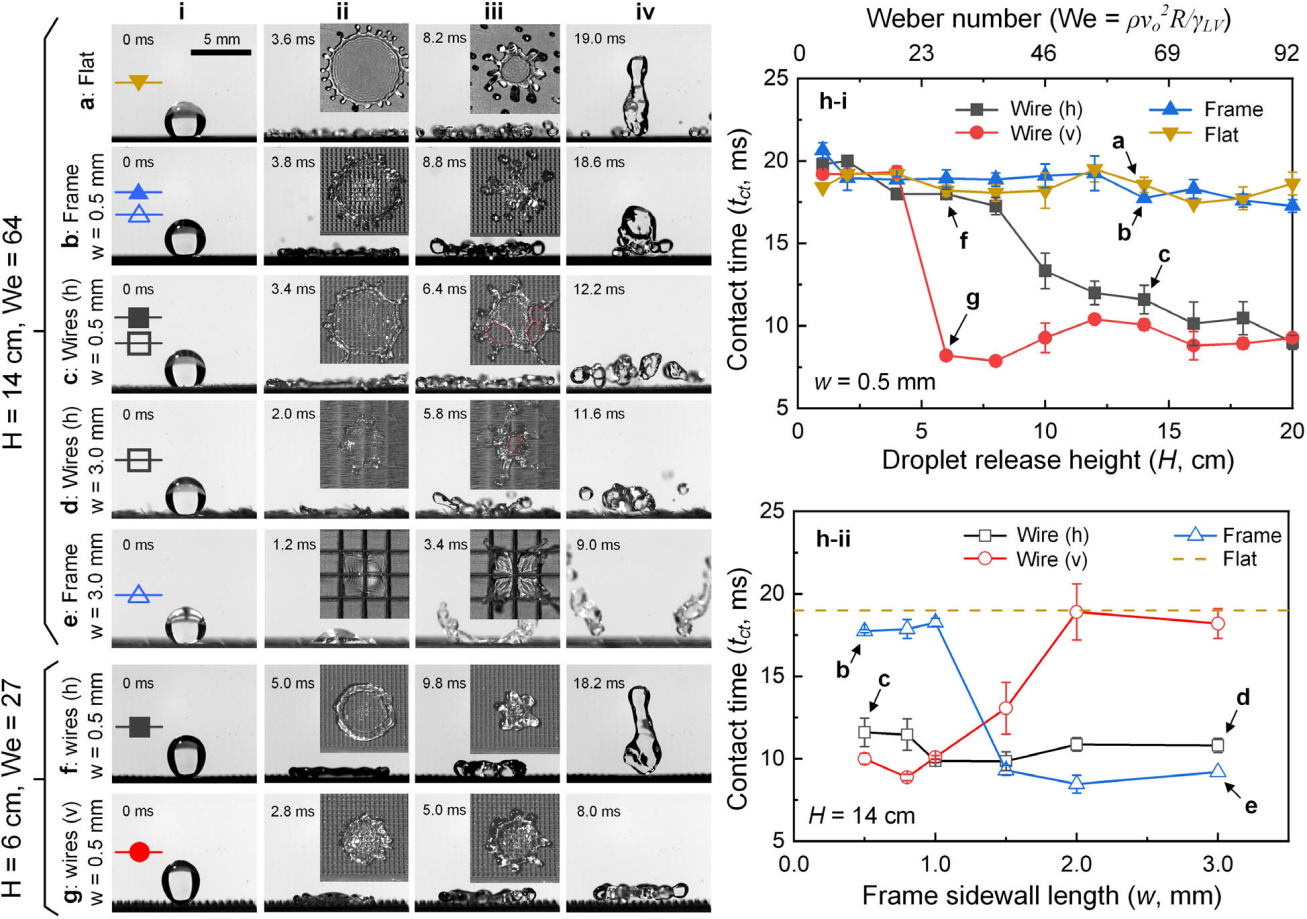
Surface characterization using droplet impact dynamics. Impact dynamics of 20 µL‐water droplets with a release height of 14 cm onto a) a superhydrophobic flat substrate, b) superhydrophobic frame arrays with frame width *w* = 0.5 mm, robust smart cilia with c) *w* = 0.5 mm and d) *w* = 3.0 mm, and e) superhydrophobic frame arrays with *w* = 3.0 mm. Impact dynamics of water droplets with a release height of 6 cm onto robust smart cilia (*w* = 0.5 mm) with f) horizontal and g) upright microwires. h) Measured droplet contact times i) with varying droplet release heights *H* (*w* = 0.5 mm) and ii) with varying frame sidewall lengths *w* (*H* = 14 cm).

The extent to which a surface repels water can also be characterized by how long an impacting droplet remains in contact with the surface, that is, the droplet‐substrate contact time *t_ct_*. Droplets (20 µL in volume) with varying release heights *H* were allowed to impact onto robust smart cilia with varying dimensions. Taking the surfaces with *w* = 0.5 mm (much smaller than the droplet size) as examples to explore the effects of *H* (**Figure** **7**h‐i), droplets exhibited constant *t_ct_* on the flat control (an example at *H* = 14 cm in Figure 7a; Video S6, Supporting Information) and frame arrays without wires (Figure 7b) despite the variations in *H*. They are well captured by the impact speed‐irrelevant inertia‐capillary time $2.4\sqrt{\rho R^{3}/\gamma_{LV}}$ ≈ 19 ms (bule and yellow dots in Figure 7h‐i).^[^ ^36^ ^]^ In contrast, once *H* exceeds 8 cm, the droplet exhibited reduced *t_ct_* on robust smart cilia with horizontal wires (*h* stands for horizontal) regardless of the variations in wire lengths (black dots in Figure 7h‐i, see also droplet impact dynamics for cases of *w* = 0.5 mm and *w* = 3.0 mm in Figure 7c,d, respectively). This reduction in *t_ct_* is not observed when *H* = 6 cm (Figure 7f). This is attributed to the rupture of the thin liquid film when a critical film thickness is locally reached,^[^ ^62^, ^63^, ^64^ ^]^ which causes the breakup of the main droplet and its earlier retraction,^[^ ^65^, ^66^ ^]^ because the horizontal wires can be considered as a flat substrate with random protrusions (microwires are not perfectly horizontal, especially under droplet impact). This observation is supported by the random occurrence and widening of holes in the spreading liquid films (iii in Figure 7c,d). At a low *H*, the droplet/film thickness is well above the critical value for film rupture, and hence no contact time reduction was observed (Figure 7f). In contrast to Figure 7f, while *H* = 6 cm and the surface (*w* = 0.5 mm) remained the same, the pancake‐like bouncing of a droplet, whose contact time *t_ct_* was reduced by more than 50%,^[^ ^36^ ^]^ was observed on the surface with upright microwires under a vertical magnetic field (*v* stands for vertical; Figure 7g and the red dots in Figure 7h‐i).

Then, to explore the effects of frame size *w* and wire length, *t_ct_* of droplets at *H* = 14 cm are plotted in Figure 7h‐ii with respect to *w*. Droplets were aimed at the intersection of frame walls to avoid the effects of impact location. All droplets exhibited reduced *t_ct_* on robust smart cilia with horizontal wires due to the thin liquid film rupture (black dots). Interestingly, a reduced *t_ct_* on frame arrays (without wires) were observed when *w* ≥1.5 mm (Figure 7e and blue dots in Figure 7h‐ii), which can be explained by the earlier film retraction caused by ridges.^[^ ^65^, ^66^ ^]^ Specifically, when four frame pockets (size of *2w*) are sufficiently larger than the droplet base, the frame walls serve as “+”‐like ridges,^[^ ^66^, ^67^ ^]^ on which the liquid film is substantially thinner than the surrounding liquids, to induce droplet breakup and hence earlier jump‐off (Figure 7e). Once *w* ≥1.5 mm is satisfied, this mechanism for *t_ct_* reduction is insensitive to frame size and hence a constant *t_ct_* was observed for varying *w* (Figure 7h‐ii). As for the cases with upright microwires, the pancake bouncing of a droplet can be observed only for *w* ≤1.0 mm (red dots). This is because longer upright microwires are prone to be deflected by impacting droplets with sufficient inertia (*H* = 14 cm), whose deflection and oscillation consume energy and hence delay droplet jump‐off.

Since horizontal microwires facilitate liquid film rupture and upright microwires under a magnetic field allow liquid downward‐to‐upward momentum redirection, the water repellency of robust smart cilia can be enhanced and actively tuned. The possible effects of substrate and/or microstructure flexibility on droplet contact time,^[^ ^68^, ^69^ ^]^ which are not evident in this study, will be investigated in the future using surfaces with similar design principles but different mechanical properties.

## 3 Conclusion

Motivated by a critical and long‐standing challenge that surfaces decorated with smart (stimuli‐responsive) and soft artificial microstructures cannot withstand mechanical damage, this study, for the first time, developed a robust ciliated surface, whose functionalities such as the on‐demand control in impact dynamics, adhesion, and transport of droplets, preserve after abrasion, dust‐related pollution, and exposition to mild acid and alkali solutions. By aligning iron‐laden aerosols perpendicularly onto vertical sidewalls of interconnected frames under a magnetic field, multilayer microwires implanted on frame sidewalls can be hidden for protection or aroused for operation in an on‐demand fashion. When facing mechanical damage, the mechanisms for surface robustness are twofold: the frames prevent the abrasion (grinding head) from contacting microwires; the lower layers of microwires are still functional although the upper layers together with the wall materials have been worn off by severe abrasion. When needed, the microwires hidden in frames re‐align uprightly under a magnetic field with their height exceeding the frames, allowing them to deliver functionalities such as reducing droplet‐substrate contact time, directional bouncing of droplets, and directional transport of droplets. Beyond this proof‐of‐concept demonstration, the generality and effectiveness of this design strategy, i.e., magneto‐responsive, flexible, superhydrophobic microwires hidden in interconnected frames, may help to push surfaces, whose functionalities rely on fragile and soft microstructures, toward real‐world applications.

## 4 Experimental Section

### Fabrication of Robust Smart Superhydrophobic Cilia

Interconnected frames (pyramidal pores) with sidewall length *w* of 0.5, 0.8, 1.0, 1.5, 2.0, and 3.0 mm, initial top wall thickness *b* of 0.1 mm, center‐to‐center spacing *λ* = *w* + *b*, and initial wall height *h*_0_ of 0.55 mm ± 0.02 mm were drilled on a 1 mm‐thick aluminum plate (16 mm × 10 mm) using a laser driller (Figure 5a‐i), followed by placing the substrate atop a cubic neodymium magnet (50 mm in width) with horizontal magnetic field lines. Unless specially noted, magnets with a strength of 450 mT were used. A mixture made with iron particles (average diameters of ≈5 *µm*), polydimethylsiloxane (PDMS) with a base‐to‐curing agent ratio of 10:1, and Toluene at a weight ratio of 3:2:6 was atomized 30 cm above the substrate at a rate of 0.03 mL s^−1^ (Figure 5a‐ii,c). The iron‐laden aerosols aligned following the magnetic field lines, forming iron‐laden PDMS microwires implanted perpendicularly on frame sidewalls, followed by heat‐curing at 120 °C for 2 h (Figure 5a‐iii). Wires were tailored to fill frame pockets with one free end, and hence, the wire length is *w*/2 controlled by the aerosol alignment time (Figure 5b). The detailed morphology and the Energy‐dispersive X‐ray Spectroscopy (EDS) of the samples were characterized by a scanning electron microscope (GeminiSEM 450, Zeiss).

A mixture of hydrophobic SiO_2_ nanoparticles (7–40 nm in diameter), 1H,1H,2H,2H‐Perfluorodecyltriethoxysilane, (3‐Aminopropyl)triethoxysilane, and ethanol at a weight ratio of 1:1:1:50 was atomized toward the surface with upright wires controlled by a magnetic field (Figure 5a‐iv), followed by heating at 80 °C for 1 h. A flat aluminum plate and substrates textured with frame arrays were also super‐hydrophobized using the same procedures.

### Characterization of Surface Durability Against Abrasion, Dust‐Related Pollution, and Chemicals

A stainless‐steel grinding head with its tip length *W* of 12 mm and a normal load *F*_↓_ of 4 and 20 N was placed on the target substrate. The substrate moved at a constant speed of 0.1 mm s^−1^ following a linear stage (OMSC60100, Red Star Yang Technology) with a travel distance per abrasion *L* of 10 mm. The robust smart cilia and the frame arrays without microwires were abraded in the same condition, whereas the maximal decrease in frame sidewall height Δ*h* was only measured for the latter case by a 3D laser microscope (OLS5100‐SAF, Olympus). A flat substrate coated with SiO_2_ nanoparticles was also abraded at *F*_↓_ of 4 N to show the mechanical damage to the SiO_2_ coatings. To have the cross‐section view of the frame pockets that house microwires, the sample was cut in half, and the boundary was located in the middle of the frame pockets. The sample was abraded as a whole and was separated after every two abrasions so that the cross‐section view images could be taken by a camera attached with a micro‐lens.

Micro‐coal ashes (S95 grade mineral powder) were deposited on the robust smart cilia to represent the dust‐like pollution.^[^ ^38^ ^]^ Such polluted surfaces were cleaned by the beating of microwires, a water jet, and the wind produced by a hand bulb ear syringe before further characterization.

To examine the chemical durability of the robust smart cilia, samples were immersed in 0.001, 0.01, 1, and 6 m HCl and NaOH solutions, corresponding to the pH values of 3, 2, 0, −0.78, 11, 12, 14, and 15, respectively, for 24 h. These samples were then characterized using the manipulation of objects and droplet impact dynamics, whose procedures were detailed below. The surface substrate was made with PDMS in place of aluminum to prevent its reaction with HCl.

### Characterization of Directional Manipulation of Objects

A magnet was placed underneath a (abraded/polluted) surface and moved at speeds ranging from 40 to 100 mm s^−1^. The moving magnet caused metachronal waves of the magneto‐responsive microwires,^[^ ^3^, ^5^ ^]^ driving a 10 µL‐water droplet or a 3 mm‐diameter polyoxymethylene (POM) sphere toward the desired direction, which was recorded by a high‐speed camera (Nova S16, Photron) at up to 1000 frames per second (fps). Alternatively, a surface with inclined microwires (the angle between the wire axial direction and the substrate bottom is *β* ≈ 30°) controlled by a magnetic field, on which a droplet experienced contrasting adhesion forces toward different motion directions, was placed atop a vibration stage with vibration frequencies in a range from 80 to 100 Hz and a fixed amplitude at 0.1 mm. The vibration‐induced droplet transport was recorded at 500 fps. By varying the sample‐magnet distance, the samples experienced magnetic field strengths varying from 50 to 300 mT. Unless specifically noted, the sample experienced a magnetic field strength of 300 mT.

### Characterization of Droplet Impact Dynamics

A water droplet with a density *ρ* of 1000 kg m^−3^, liquid‐vapor interfacial tension γ_*LV*_ of 0.072 N m^−1^, a volume of 20 µL was released at heights *H* varying from 0.01 to 0.2 m, corresponding to droplet impact speed *v*_0_ ranging from 0.44 to 2 m s^−1^ and Weber number *We* ranging from 4.5 to 92. Droplet impact dynamics were visualized by two synchronized high‐speed cameras (Nova S16, Photron) at 5000 fps from side and top views to ensure the droplet impacted at the intersection of frame walls for *w* ≥1.5 mm. The droplet‐substrate contact time was measured as the time interval from droplet‐substrate contact to their complete separation and the average of at least three reproducible results was reported.

### Characterization of Droplet Lateral Adhesion Force and Receding Contact Angles

The surface was mounted on a linear stage moving at a speed of 0.1 mm s^−1^. Water droplets with volume of 20 and 40 µL were deposited on the surface with its side attached to a hydrophilic patch (1.5 mm in diameter) at the free end of a superhydrophobic copper beam (calibrated spring constant *k_beam_* of 220 mN m^−1^). The pinned droplet on the surface, which moved together with the linear stage, pulled the copper beam, resulting in its tip deflection δ measured by a displacement sensor at 100 Hz. The droplet adhesion force *k_beam_* δ was hence measured with respect to surface displacement. The droplet side and back views were recorded by two cameras (D5600, Nikon) at 60 fps to visualize the droplet shape profile and the detailed droplet‐wire interaction. By increasing and decreasing droplet volume at a rate of 0.1 µL s^−1^, the droplet advancing and receding contact angles were measured respectively by a goniometer (OCA25, Dataphysics) under room conditions.

## Conflict of Interest

The authors declare no conflict of interest.

## Supporting information



Supplemental Video 1

Supplemental Video 2

Supplemental Video 3

Supplemental Video 4

Supplemental Video 5

Supplemental Video 6

## Data Availability

The data that support the findings of this study are available from the corresponding author upon reasonable request.
